# A Classification of Fitness Components in Elite Alpine Skiers: A Cluster Analysis

**DOI:** 10.3390/ijerph20105841

**Published:** 2023-05-17

**Authors:** Gabriella Penitente, Hayden A. Young, William A. Sands, Jeni R. McNeal

**Affiliations:** 1 Academy of Sport and Physical Activity, Sheffield Hallam University, Sheffield S10 2BP, UK; 2 US Ski and Snowboard Association, Park City, UT 84060, USA; 3 Department of Physical Education, Health and Recreation, Eastern Washington University, Cheney, WA 99004-2431, USA

**Keywords:** winter sports, aerobic capacity, anaerobic capacity, explosive force, performance profile, dendrogram

## Abstract

The current study is an exploratory, secondary data analysis of a selection of physiological and biomechanical fitness components used to assess elite alpine skiers. The present study will provide new knowledge that can be used to aid training prescription and talent identification. A hierarchical cluster analysis was used to identify groups of variables that are crucial for elite alpine skiers and differences based on sex and competition level. The key findings of the study are the patterns that emerged in the generated dendrograms. Physiological and biomechanical fitness components are differentiated in the dendrograms of male and female world-cup-level alpine skiers, but not in non-world-cup athletes. Components related to the aerobic and anaerobic capacity tightly cluster in male athletes at world cup and non-world-cup level, and female world cup athletes. Lower body explosive force production appears to be more critical in male world cup athletes than female world cup athletes. More research is needed into the importance of isometric strength in the lower body. Future research should use larger sample sizes and consider other alpine ski demographics.

## 1. Introduction

Alpine ski racing has been part of the Winter Olympics since 1936. It traditionally consists of two speeds and two technical events, which vary in turns, terrain, and jumps (Gilgien et al., 2018), but all use gravity as the driving force [1]. Downhill and super-giant slalom are considered the speed events, reaching speeds of up to 130 km/h, lasting 2–3 and 1–2 min, respectively. Slalom and giant slalom are technical events and reach speeds of approximately 20–60 km/h. The technical events typically last 45–90 s, occur on steeper terrain, and include short and narrow turns [2]. 

All alpine ski events require physical and technical competence [3], meaning no distinct feature can identify the potential for success [4]. Small winning margins (often less than half a second) highlight the need for a deep understanding of the components of fitness that influence alpine ski performance [5]. Furthermore, Nygaard et al. [6] determined that the technical aspect of skiing makes “time on the snow” a critical factor in athlete development. During periods of intense competition, physical and technical training time can become limited due to a lack of training facilities, time, or finance. It becomes vital that the planned training is evidence-based and relevant to develop the crucial components of fitness and minimise wasted effort [4,7].

Lower body strength and power are crucial performance components for elite skiers [8,9,10]. Over the years, the improvements made to ski equipment have made alpine skiing more dynamic; carved turns have become tighter, which has increased the centrifugal force placed on the lower bodies of ski racers [10]. Repeated sharp turns and high accelerations have also increased the intensity of the load on the athletes’ lower body, as well as the risk of injury [11]. Approximately 37% of elite alpine skiers are injured per season; anterior cruciate ligament (ACL) injuries are the most frequent diagnosis, but other legs and lower back injuries are also common [12]. The injury rate and type are roughly equal between alpine ski disciplines [13]. A high level of physical fitness has been identified as a crucial component for skiers to improve their performance and prevent injuries [14]. The ability to generate a high level of maximum strength allows athletes to work at a reduced percentage of their maximum voluntary contraction, enabling them to safely sustain high-intensity loads [14].

A unique feature of alpine skiing is the continuous vertical displacement of body mass by which the skier generates speed and kinetic energy [15]. Leg extensor muscles are primarily activated during the turning phase, and the muscular load is caused by an accelerative force proportional to the weight and speed of the skier and inversely proportional to the radius of the turn [15]. A larger capacity for eccentric force improves the ability of the skier to maintain turn speed throughout the race [15]. Emphasis is placed on the athlete’s explosive and dynamic strength capacity due to the repeated, high velocity turns that require muscles to generate high forces within a few seconds [16,17].

The velocity of a skier is influenced by the balance of external forces, including gravity, ground reaction force from the snow surface, and aerodynamic drag. The latter can account for as much as 90% of the total resistive forces acting on a skier, any reduction in this drag can significantly improve performance [18]. Skiers utilise the tucked position to minimise aerodynamic drag and maximise velocity in straight sections of the ski courses; the ability to hold this position is crucial to performance. The tucked position requires isometric contraction at the hip, engaging the hamstring and quadricep muscles [16]. Therefore, a high level of isometric strength is necessary for these muscles to sustain the tucked position for the race duration [16]. 

The intensity and short duration of most alpine ski races [19], coupled with the hypoxic conditions at high altitude [20], make this sport predominantly an anaerobic discipline that engages both anaerobic pathways, the adenosine triphosphate–phosphocreatine (ATP–PCr) system and the anaerobic glycolysis system [19]. Scientists found the high-energy and environmental demands of alpine skiing result in an exaggerated reliance on anaerobic energy production and reported that metabolic demand could be as much as 200%, highlighting its importance to alpine skiers [21,22,23].

There is considerable disagreement in the literature regarding the direct impact of aerobic capacity on alpine ski performance. For example, White and Johnson [24] suggest aerobic capacity cannot be used to discriminate alpine skiers of varying ability, but Neumayr et al. [25] found high maximal and submaximal aerobic capacity to be strongly correlated with international ski success in the Austrian national team. Some of the variance in results has been attributed to differences in testing; some testing uses a cycle ergometer, whilst others use a treadmill [3]. Despite the disagreement on the direct impact of aerobic capacity, it has been suggested that there are indirect impacts worthy of note. An efficient aerobic system is essential for recovery between runs during competition and training and to support the high energy requirements of long and exhausting competitions and on-snow training sessions [25].

Whilst some studies into the physiological, technical, and injury aspects of alpine skiing exist, more up-to-date evidence-based knowledge is required to match the fast development of the sport [26]. Scientists suggest that a deeper understanding of the effects of training on the physical readiness of skiers is needed to inform training and recovery strategies [4,7,13,25]. Furthermore, a better understanding of the fitness components required for elite alpine ski racers is necessary to facilitate future performance enhancement, injury prevention, talent identification, and training prescription. Muscular forces and energy systems were highlighted as a particular area of interest [3].

The current study is an exploratory, secondary data analysis of selected physiological and biomechanical fitness components used to assess elite alpine skiers. A series of hierarchical cluster analyses identify variables that are crucial for all elite alpine skiers and differences based on competition level and sex. This analysis will aid coaches in optimising training prescriptions for elite alpine ski racers, aid in talent identification, and guide future research in the field.

## 2. Materials and Methods

### 2.1. Participants

This study was based on archived data collected and stored by United States Ski and Snowboard Association (USSA) sport science team between 27 January 2010 and 7 October 2015. All names and personal identification were removed, and only group data were used for the analysis.

The original data set contained 134 participants affiliated with the USSA national team, 85 males (29 world cup and 56 non-world cup) and 49 females (19 world cup and 30 non-world cup). Athletes were classified as “world cup” or “non-world cup” based on whether they competed at a world-cup-level competition within the six-year duration of the data collection. All athletes competed in one or more alpine ski disciplines.

Eastern Washington University was previously awarded ethics for the primary data collection. Five testing batteries were conducted per year on alpine skiers affiliated with the USSA national team [27]. The original data set contained nearly 1500 variables, including sex, age, and competition level, as well as results measured or calculated from the testing batteries relating to anthropometry, body composition, strength, power, and other physiological fitness components. Missing data and non-participation resulted in large gaps in longitudinal time-series data and many incomplete test results; missing data ranged from around 5% to 80% per variable [27]. 

Due to varying amounts of missing data, and based on the recommendations of previous research, the following tests from the original data set were selected for analysis:

1. Incremental Cycle Ergometer Test

The incremental cycle ergometer test consists of any number of five-minute stages with constant workloads during each stage. The workloads increase by 40 W at the beginning of each stage with males starting at 80 W and females starting at 40 W. The test continues until the athlete reaches or exceeds a blood lactate concentration of 4.0 mmol.L^−1^ [27]. A cycling pace of around 80–90 rpm is maintained by the athlete. The test was conducted on a LodeTM electronically braked ergometer, enabling the workload to be held constant with varying pedalling cadences [27]. The variables collected from this test and used in the current study were heart rates (bpm) and workloads (W) at 2, 3, and 4 mmol.L^−1^ blood lactate concentrations and related to submaximal aerobic and anaerobic capacity.

2. Strength Tests

The isometric squat, squat jump, and countermovement jump test were used to assess different components of strength [27]. The isometric squat test requires that athletes stand on a force platform below an immoveable horizontal bar, placed above the posterior deltoids at the base of the neck. Athletes stand with their feet shoulder-width apart, knees bent, and trunk almost vertical. When instructed, athletes push against the ground as hard as possible. A one-dimensional force platform was used to detect and record vertical ground reaction force [27]. Two trials were performed, and the mean was calculated. Relative strength (kg/body mass) was calculated using the athletes’ body mass and results from the isometric squat. Squat and countermovement jumps were conducted on an AMTI force platform sampling at 1000 Hz [27]. The squat jump is performed by the athlete lowering themselves to the bottom position of their jump, pausing, and then jumping as high as possible. The countermovement jump involves starting in a standing position, dropping down quickly and immediately jumping as high as possible. Both jumps require the arms to maintain a neutral position, and the legs to remain extended after take-off. Peak force (N), power (W), displacement (cm), and velocity (m.s^−1^) were measured using the force platform. Countermovement jump relative power (W/body mass) was also calculated, using power (W) and the athletes body mass.

### 2.2. Data Screening

First, the original six-year data set was trimmed to only contain data from the incremental cycle ergometer and strength tests. For each year of testing, athletes with missing or erroneous data in any of the variables from the incremental cycle ergometer or strength tests were removed from the data set. Each of the tests was compared to identify the one with the most data. The fourth year of testing (2013) contained the most athletes with complete data sets in the incremental ergometer cycle and strength tests, so it was selected for analysis. 

### 2.3. Participants: Post-Screening

Post-screening, the data set was split based on sex and competition level. In total, there were 45 athletes, 28 males (14 world cup and 14 non-world-cup) and 17 females (12 world cup and 5 non-world-cup). Male world cup athletes had a mean age of 24 ± 3 years and mean body mass of 84.50 ± 4.04 kg, whilst male non-world-cup athletes had a mean age of 20 ± 2 years and mean body mass of 86.82 ± 8.97 kg. Female world cup athletes had a mean age of 23 ± 3 years and a mean body mass of 70.65 ± 4.29 kg, whilst female non-world-cup athletes had a mean age of 19 ± 1 years and a mean body mass of 69.79 ± 5.26 kg. 

### 2.4. Statistical Analysis

Each variable was found to be normally distributed using the Shapiro–Wilk test for normality and/or visual inspection of the bell curves and box plots relating to each variable. All variables were reported as mean ± standard deviation for each sex and competition level group. Variables were then transformed into the zeta score to analyse the presence of outliers and negate the effects of different measuring scales. A series of hierarchical cluster analyses were used to cluster the variables for each sex and competition level group. As a measure of similarity, the squared Euclidean distances agglomeration technique was used. Ward’s clustering method was used, and the data were grouped by variable. Each hierarchical cluster analysis generated a dendrogram, which is inspected below. All statistical analysis was performed using SPSS software (IBM SPSS Statistics Version 26).

## 3. Results

### 3.1. Descriptive Statistics

Descriptive statistics were calculated using SPSS software. Table 1 and Table 2 provide the means (±standard deviation), minimum, and maximum values for the six variables (top six) from the incremental cycle ergometer test and ten variables (bottom ten) from the various strength tests, for both male and female, world cup and non-world-cup alpine skiers.

### 3.2. Dendrograms

The dendrograms generated by the hierarchical cluster analysis of fitness components in the four testing groups (world cup males, world cup females, non-world-cup males, and non-world-cup females) are provided and described below.

### 3.3. World Cup Males

**Figure 1:**
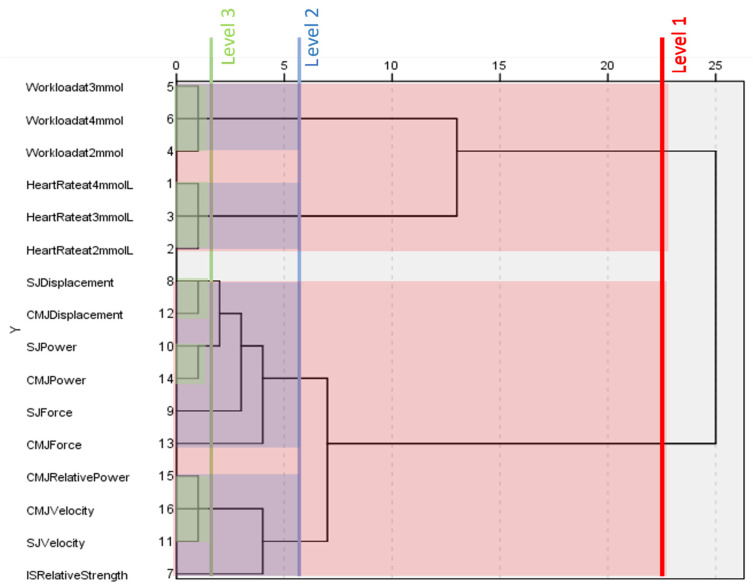
Dendrogram generated by a hierarchical cluster analysis of testing conducted on male world cup alpine skiers. Clusters present at different levels are highlighted. HR = heart rate at given blood lactate concentration, WL= workload at given blood lactate concentration, IS = isometric squat, SJ = squat jump, CMJ = countermovement jump, Rs = relative strength, D = vertical displacement, F = force, P = power, Rp = relative power, v = velocity.

Figure 1 shows the fitness component classification generated by the hierarchical cluster analysis of male, world-cup-level alpine skiers. This dendrogram can fully differentiate between the physiological and biomechanical fitness components. At level one, two large clusters form; the first contains all variables from the incremental cycle ergometer test (physiological variables) and the second contains all variables from the various strength tests (biomechanical variables), no outliers are present at this level.

At level two, the dendrogram splits further into four distinct clusters. Two of the four clusters present here separate the heart rate variables from the workload variables at all three blood lactate concentrations. The next two clusters are able to distinguish variables measuring displacement, power, and force, from those measuring isometric strength, relative power, and velocity. 

At level three, workload and heart rate variables maintain their clusters from level two, whereas variables relating to displacement and power form their own new clusters. Relative power clusters with both measures of velocity. Isometric strength, squat jump force, and countermovement jump force do not cluster with any other variables at level three. Despite this, it appears that squat jump force and countermovement jump force are closely related to the measures of displacement and power in squat and countermovement jumps.

### 3.4. World Cup Females

**Figure 2:**
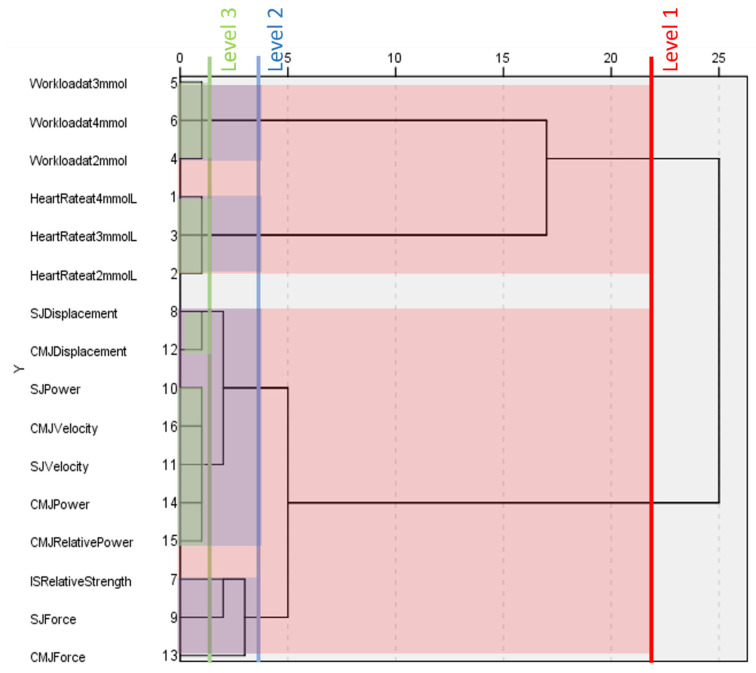
Dendrogram generated by a hierarchical cluster analysis of testing conducted on female world cup alpine skiers. Clusters present at different levels are highlighted. HR = heart rate at given blood lactate concentration, WL= workload at given blood lactate concentration, IS = isometric squat, SJ = squat jump, CMJ = countermovement jump, Rs = relative strength, D = vertical displacement, F = force, P = power, Rp = relative power, v = velocity.

Similar to Figure 1, Figure 2 shows that the dendrogram generated by the hierarchical cluster analysis of female world cup alpine skiers can fully differentiate between physiological and biomechanical components of fitness. At level one, two large clusters form, separating variables related to the incremental ergometer cycle test and the various strength tests.

At level two, four more clusters form. The first two clusters that form at level two are identical to those that form at the same level in the male world cup dendrogram; workloads and heart rates are separated for all three blood lactate concentrations. The next cluster that forms at level two groups variables relates to displacement, power, and velocity. The final cluster at level two groups both measures of force with relative strength in the isometric squat. 

Level three in this dendrogram reveals four distinct clusters. Again, workloads and heart rate variables retain their clusters from level two. Power and velocity from both the squat jump and countermovement jump form one large cluster, while displacement from both jumps form their own. Isometric squat relative strength and force variables do not cluster with any other variables at this level. 

### 3.5. Non-World-Cup Males

**Figure 3:**
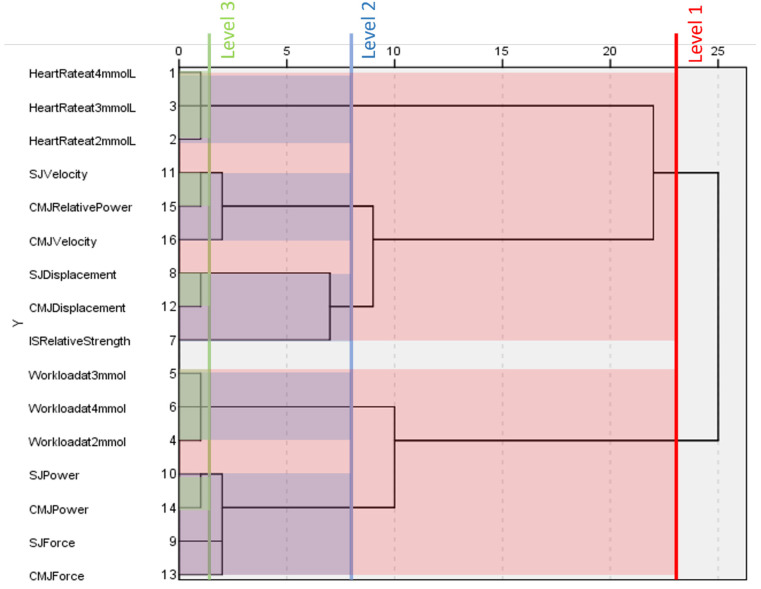
Dendrogram generated by a hierarchical cluster analysis of testing conducted on male non-world-cup alpine skiers. Clusters present at different levels are highlighted. HR = heart rate at given blood lactate concentration, WL = workload at given blood lactate concentration, IS = isometric squat, SJ = squat jump, CMJ = countermovement jump, Rs = relative strength, D = vertical displacement, F = force, P = power, Rp = relative power, v = velocity.

Figure 3 shows the fitness component classification for non-world-cup males. This dendrogram is different from world cup males and females, as it is unable to separate all physiological and biomechanical fitness components at level one. Alternatively, variables relating to workload at different blood lactate concentrations, force, and power cluster together. The remaining variables, heart rate at different blood lactate concentrations, displacement, velocity, relative power, and isometric strength, cluster together.

At level two, workload and heart rate at different blood lactate concentrations form their own clusters. Power and force in the squat jump and countermovement jump continue to cluster together. Velocity and countermovement jump power separate from displacement and isometric strength variables. 

Several variables do not cluster at level three; force, isometric strength, and countermovement jump velocity do not cluster with any other variables. Workload and heart rate at different blood lactate concentrations maintain their clusters from level two. Measures of jump power form a tighter cluster, as do measures of displacement. Countermovement jump, relative power, and squat jump velocity cluster together at this level.

### 3.6. Non-World-Cup Females

**Figure 4:**
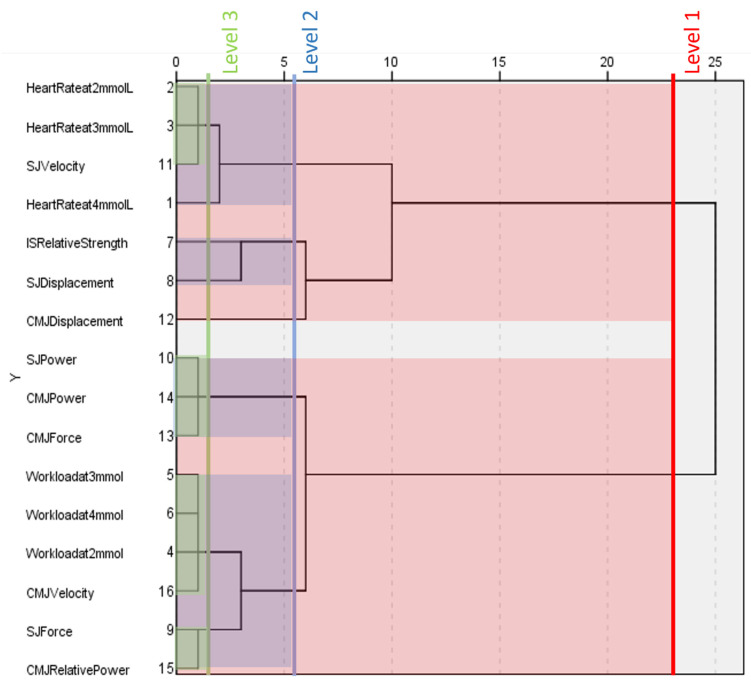
Dendrogram generated by a hierarchical cluster analysis of testing conducted on female non-world-cup alpine skiers. Clusters present at different levels are highlighted. HR = heart rate at given blood lactate concentration, WL= workload at given blood lactate concentration, IS = isometric squat, SJ = squat jump, CMJ = countermovement jump, Rs = relative strength, D = vertical displacement, F = force, P = power, Rp = relative power, v = velocity.

The final dendrogram, shown in Figure 4, is also unable to separate all physiological and biomechanical components of fitness at level one. The dendrogram shows that heart rate at different blood lactate concentrations, isometric strength, displacement, and squat jump velocity cluster together at level one. The second cluster at level one contains variables relating to power, force, workload at different blood lactate concentrations, relative power, and countermovement jump velocity.

Level two reveals a further four clusters, none of which follow the patterns of the previous dendrograms. Firstly, heart rate at different blood lactate concentrations clusters with squat jump velocity, while workload at different blood lactate concentrations clusters with countermovement jump velocity, relative power, and squat jump force. This is the only dendrogram in which heart rates and workloads do not form their own clusters. Measures of maximal power group with countermovement jump force. Isometric strength clusters with measures of displacement.

Finally, level three highlights four clusters and four variables that do not cluster. In contrast to previous dendrograms, heart rate at 4 mmol.L^−1^ blood lactate concentration does not cluster with the other heart rate measurements and is an outlier at this level instead. Heart rate at 2 and 3 mmol.L^−1^ blood lactate concentrations clusters with squat jump velocity. All workload measurements at different blood lactate concentrations cluster together with countermovement jump velocity. Measurements of maximal power and countermovement jump force retain their cluster from level two. Squat jump force clusters with countermovement jump relative power, while measurements of displacement and isometric strength do not cluster at this level.

## 4. Discussion

The present study is an exploratory, secondary data analysis aiming to classify a selection of physiological and biomechanical fitness components used to assess elite alpine skiers. To the best of the knowledge of the authors, this is the first study to classify the fitness components of alpine skiers in this way and aims to build on existing knowledge to aid in the training prescription and talent identification of elite alpine skiers, and guide future research in the field. The key findings of the current study are the patterns that emerge in the dendrograms generated when classifying fitness components in different groups of elite alpine skiers.

Firstly, no outliers are present in any of the four dendrograms at level one. This indicates that all the fitness components analysed are somewhat related. This seems intuitive, given the similarity in the athletes’ ability level in each group, and, therefore, the performances in each of the tests conducted.

The generated dendrograms display a clear differentiation between all physiological and biomechanical fitness components in both male and female world-cup-level athletes. This clear differentiation is not a feature in the dendrograms of non-world-cup males and females. Furthermore, the clustering displayed for the physiological variables (workloads and heart rates) is identical between male and female world cup athletes. Hierarchical cluster analyses cluster variables based on similarity, indicating that all the fitness components that cluster together share a certain level of similarity. Given that the variables involved in the study were converted to zeta score to negate the effects of their units, one interpretation could be that the similarity between clustered variables is due to the importance of the component to the performance of an alpine skier. This would mean that all fitness components analysed have some relationship with the performance of world-cup-level alpine skiers. This interpretation is consistent with previous research, which suggests that a high level of all-round physical fitness is essential for elite alpine skiers. Alpine skiing is a multifaceted sport that is not extreme in terms of the physical demands on the body, meaning a wide variety of fitness components can be indicative of elite alpine skiers [3]. Moreover, highly trained strength, power, aerobic and anaerobic capacity, balance, and coordination have all been found in elite alpine skiers [13], highlighting how no one feature can differentiate the elite athletes from the non-elite [4].

In male and female world cup athletes, heart rate measurements at various blood lactate concentrations cluster very tightly, only separating at level three of the dendrogram, as do the workload measurements at varying blood lactate concentrations. Both variables were measured on the incremental cycle ergometer test and related to the capacity of the aerobic and anaerobic energy systems to produce power. Blood lactate accumulates due to muscle activation and coincides with the onset of muscular fatigue [28]. Heart rate increases proportionally to clear the blood lactate and avoid muscle acidosis, which impairs performance. The point at which blood lactate production surpasses the clearance rate (4 mmol.L^−1^) is termed the anaerobic threshold. It marks the point at which the body becomes reliant on anaerobic energy systems [29]. A lower heart rate and higher workload at the submaximal exercise intensities in the present study indicate a higher aerobic and anaerobic capacity [29]. The tight clusters in both male and female world cup athletes at all blood lactate concentrations for heart rate and workload could indicate the importance of the aerobic and anaerobic metabolic pathways to elite alpine skiers. The findings of Ferguson, Spörri et al. [21,22], and White and Wells [23] found the anaerobic capacity to be critical to elite alpine skiing. Given that alpine skiing predominantly utilises the anaerobic pathway [19], it would seem logical that a high anaerobic capacity would indicate elite alpine skiers. 

On the other hand, the current findings disagree with those of White and Johnson [24], who found that aerobic power is not as important to alpine skiing. The current hierarchical cluster analysis is unable to differentiate between the heart rates and workload at given submaximal aerobic (2 and 3 mmol.L^−1^ blood lactate concentrations) and anaerobic (4 mmol.L^−1^ blood lactate concentrations) exercise intensities, and this could indicate that aerobic capacity is also important to elite skiers. Despite the current study and others [3] suggesting the importance of aerobic capacity of elite alpine skiers, it has been proposed that this is a side effect of the high training load common for alpine ski racers, and not a fitness component especially important to enhancing the performance of alpine skiers [25].

The key difference between the dendrograms generated for male and females at world-cup-level relates to the biomechanical variables. In male world cup athletes, squat and countermovement jump force show a closer relationship with power and displacement of the same jumps than in female world cup skiers. Both the countermovement jump and squat jump are measures of lower body explosive strength [30]. This finding could indicate that explosive force production is more important to male world cup athletes than female world cup athletes. It has previously been determined that explosive strength is critical to success in alpine skiing due to its importance in counteracting the centrifugal forces placed on skiers whilst turning at high velocity [16,17]. The velocity of a skier is influenced by the balance of external forces, one of which is gravity [18]. The force of gravity acting on an object is a product of the object’s mass and the gravitational acceleration constant (approximately 9.81 m.s^−2^). Thus, assuming the other forces acting on the skiers are approximately equal, the skier’s velocity approaching a turn is proportional to their body mass. A study by Hogstrom et al. [31] found statistically significant differences in the body mass of male and female elite skiers. This higher body mass could suggest that male alpine skiers experience higher velocities and higher centrifugal forces while turning. This would mean the ability of skiers to generate explosive force is more important to male athletes to turn effectively and avoid injuries due to high centrifugal force. 

Following force, the clustering of power variables from countermovement and squat jumps are very similar in males and females at the world cup level. Maximal power has consistently been shown to be predictive of successful alpine ski performance at the elite level [16,32] and jump tests have been shown to successfully determine maximal power in alpine ski racers, as they attempt to simulate the physical demands of ski racing [30]. Maximal power reflects the power developed during all out, short-term effort and indicates the energy output capacity of the muscles [10]. Neumayr et al. [25] found that beyond a certain point, power is not a determining factor of alpine ski performance, and this could mean that maximal power output is more relevant to avoiding injury, but further research is needed. Irrespective of the reasoning, maximal power output appears to be similarly important to males and females at world cup level.

At level three, isometric strength does not cluster with any other variables in any of the four dendrograms. This could mean that it is not a crucial component of fitness in alpine skiing. A high level of isometric strength allows the skier to maintain the tucked position where necessary for the duration of the race [16], reducing aerodynamic drag [18]. Despite this, other fitness components such as core strength have also been important for maintaining the tucked position [16]. This means that while lower body isometric strength may be somewhat important, it may not be one of the more crucial fitness components to alpine skiers. It is also possible that the lack of clustering for this variable results from the testing type. Isometric strength was the only component of strength that was not measured using a jump test. This may have led to the variable becoming an outlier. Further research is needed to determine the importance of lower body isometric strength.

Before discussing the female non-world-cup group results, it is important to note the relatively small sample size. This group contained only five athletes from the 45 athlete sample, so the results from the hierarchical cluster analysis of this group must be interpreted with caution. Non-world-cup females are the only group in which the heart rate variables do not all cluster together. As these variables are found to cluster in world cup females, this could indicate that aerobic and anaerobic capacity is a critical fitness component to train in non-world-cup female athletes. However, with a larger sample size it is very possible that non-world-cup females would display similar cluster patterns to non-world-cup males.

Cluster analysis is a statistical technique that aims to classify data points into groups based on their similarity, while dendrograms provide a visual representation of the hierarchical structure of these groups. In sport science, hierarchical clustering has been used to provide valuable insights into patterns and relationships among performance variables [33], grouping athletes based on their physical and physiological characteristics [34], and clustering individuals based on their movement patterns [35,36]. In the context of sport talent identification and training design, identifying the relevant variables is essential to achieving functional data use. This is because the success of talent identification and training programs is highly dependent on the accuracy and validity of the data used to inform them [37,38,39,40]. When designing a talent identification program, it is essential to identify and prioritise the variables that are most relevant to the sport in question. Similarly, when designing a training program, identifying the most relevant variables can help coaches to tailor their interventions to the specific needs and abilities of their athletes.

As this was the first study of its kind in world-class alpine skiers, it was unknown whether this type of analysis would result in any useful output. As a result, only a small selection of fitness components were selected for analysis in this study. The small selection of fitness components could mean that each has an exaggerated influence over the hierarchical cluster analysis and, therefore, the resulting dendrogram that formed. This may have caused results that are not truly representative of an alpine skier. 

The variables selected for use in this study led to very small sample sizes (male world cup = 14, male non-world-cup = 14, female world cup = 12, female non-world-cup = 5), particularly in non-world-cup females. Other studies on alpine ski racing have also acknowledged the limitation of small sample sizes when working with world-class athletes [10,41]. The small sample size could mean the results of this study are not representative of the alpine ski racing population. Furthermore, athletes involved in this study were all affiliated with the USSA national team. Training programs and practices specific to these athletes could affect the testing results, again meaning the results may not be representative.

Finally, the visual inspection of the dendrograms is slightly subjective. This means different researchers could have very different interpretations of the meaning behind each dendrogram. The author has highlighted possible interpretations but acknowledges that there are likely to be other possible interpretations. A framework may be necessary to standardise the interpretation of dendrograms generated through the classification of fitness components of elite alpine skiers if they are to be used regularly for research purposes.

## 5. Conclusions

Overall, the results of this study suggest the classification of fitness components can be used to further understand the fitness components relevant to alpine ski performance.

Some elements of the dendrograms generated show remarkable similarity whilst other elements differ between the sex and competition level groups analysed. Key similarities are found between the males and females at the world cup level, and a few insightful differences are highlighted between world cup males and females and their respective non-world-cup counterparts. World-cup-level male and female athletes display a greater order level in their dendrograms, while non-world-cup dendrograms appear more chaotic. Aerobic and anaerobic fitness components appear to be similarly important to world-cup-level males and females, but components of explosive strength could be more critical to males. The clustering of maximal power is also very similar in males and females, but isometric strength does not seem to be closely related to any of the other fitness components analysed.

The clear differentiation of physiological and biomechanical fitness components by the hierarchical cluster analysis appears to identify both male and female world-cup-level alpine skiers and, thus, could be helpful in talent identification. The analysis also identifies other components that could be useful for training prescription and talent identification. However, due to the small sample sizes and lack of supporting research on this subject, further research is needed. Future research could focus on expanding the knowledge base in this area by using hierarchical cluster analysis to assess a larger sample of athletes and a larger selection of fitness components. Body composition measures, functional movement screens, and other components of fitness could be included. Furthermore, future studies could analyse how fitness components of specific age groups or other competition levels cluster, such as adolescent or novice-level skiers. The results of our study demonstrate the value of hierarchical clustering for functional data in simplifying the variable selection process needed for sport talent identification and training design. Finally, if hierarchical cluster analysis of fitness components becomes common practice. a framework should be developed to standardise the interpretation of the dendrograms and eliminate the subjective nature of the visual inspection.

## Figures and Tables

**Table 1:** Descriptive statistics recorded (provided as mean, standard deviation, minimum, and maximum) for male world cup and non-world-cup alpine skiers.

	Male World Cup (*n* = 14)		Male Non-World-Cup (*n* = 14)	
	Mean ± SD	Min–Max	Mean ± SD	Min–Max
HR_2 mmol.L_^−1^ (bpm)	147.77 (9.90)	137–165	149.11 (16.99)	119–169
HR_3 mmol.L_^−1^ (bpm)	158.91 (8.52)	146–174	161.48 (14.93)	132–178
HR_4 mmol.L_^−1^ (bpm)	165.57 (8.35)	153–182	168.72 (14.18)	139–186
WL_2 mmol.L_^−1^ (W)	204.63 (30.06)	158.5–245	186.89 (24.40)	156.67–235.33
WL_3 mmol.L_^−1^ (W)	239.04 (30.45)	194.33–289.00	226.14 (27.01)	185.00–269.33
WL_4 mmol.L_^−1^ (W)	262.59 (30.32)	214.67–312.00	251.51 (28.02)	209.00–294.00
IS_Rs_ (kg/body mass)	2.95 (0.22)	2.50–3.22	2.76 (0.16)	2.53–3.01
SJ_D_ (cm)	48.16 (4.73)	40.33–54.25	44.72 (4.54)	37.18–56.45
SJ_F_ (N)	2024.55 (188.72)	1698–2403.25	1978.87 (246.49)	1535.50–2476.50
SJ_P_ (W)	3267.57 (310.18)	2575.75–3707.00	3354.67 (370.08)	2738.75–3892.67
SJ_V_ (m.s^−1^)	2.51 (0.12)	2.29–2.71	2.51 (0.15)	2.18–2.77
CMJ_D_ (cm)	53.80 (5.42)	42.72–62.95	50.40 (4.51)	45.45–64.45
CMJ_F_ (N)	2336.14 (240.74)	2027.25–2788.00	2230.78 (296.52)	1550.50–2710.67
CMJ_P_ (W)	3642.24 (407.51)	2851.75–4585.00	3820.25 (404.03)	2991.50–4267.33
CMJ_Rp_ (W/body mass)	2.71 (0.16)	2.43–3.04	2.69 (0.15)	2.34–2.91
CMJ_V_ (m.s^−1^)	2.70 (0.15)	2.40–2.95	2.72 (0.16)	2.40–3.03

HR = heart rate _at given blood lactate concentration_, WL= workload _at given blood lactate concentration,_ IS = isometric squat, SJ = squat jump, CMJ = countermovement Jump, _Rs_ = relative strength, _D_ = vertical displacement, _F_ = force, _P_ = power, _Rp_ = relative power, _v_ = velocity.

**Table 2:** Descriptive statistics recorded (provided as their mean, standard deviation, minimum, and maximum) for female world cup and non-world-cup alpine skiers.

	Female World Cup (*n* = 12)		Female Non-World-Cup (*n* = 5)	
	Mean ± SD	Min–Max	Mean ± SD	Min–Max
HR_2 mmol.L_^−1^ (bpm)	146.99 (7.24)	137–158	150.70 (8.70)	140–163
HR_3 mmol.L_^−1^ (bpm)	159.54 (6.93)	150–170	160.53 (9.15)	149–173
HR_4 mmol.L_^−1^ (bpm)	167.81 (5.91)	158–177	169.60 (6.67)	164–180
WL_2 mmol.L_^−1^ (W)	146.11 (17.04)	113–176	142.00 (25.27)	111.00–177.00
WL_3 mmol.L_^−1^ (W)	176.10 (17.11)	149–207	171.70 (29.21)	137.50–213.33
WL_4 mmol.L_^−1^ (W)	194.57 (17.39)	170.67–225.00	190.57 (28.41)	161.00–231.67
IS_Rs_ (kg/body mass)	2.49 (0.23)	2.21–2.84	2.40 (0.24)	2.16–2.76
SJ_D_ (cm)	33.32 (3.87)	24.67–37.48	35.05 (2.61)	31.67–37.58
SJ_F_ (N)	1550.15 (139.36)	1313.25–1785.50	1592.73 (127.87)	1462.00–1773.83
SJ_P_ (W)	2222.31 (322.81)	1655.00–2781.00	2363.63 (359.59)	1988.00–2780.00
SJ_V_ (m.s^−1^)	2.11 (0.16)	1.83–2.35	2.18 (0.09)	2.09–2.31
CMJ_D_ (cm)	36.53 (5.00)	26.15–43.02	36.44 (3.63)	30.55–40.10
CMJ_F_ (N)	1675.01 (184.32)	1402.25–2007.67	1649.17 (219.77)	1362.00–1880.50
CMJ_P_ (W)	2523.44 (370.60)	1851.25–3002.25	2624.30 (431.36)	2139.00–3035.00
CMJ_Rp_ (W/body mass)	35.66 (4.50)	29.19–41.88	37.04 (3.66)	31.55–41.71
CMJ_V_ (m.s^−1^)	2.30 (0.21)	1.96–2.60	2.38 (0.12)	2.26–2.56

HR = heart rate _at given blood lactate concentration_, WL= workload _at given blood lactate concentration_, IS = isometric squat, SJ = squat jump, CMJ = countermovement jump, _Rs_ = relative strength, _D_ = vertical displacement, _F_ = force, _P_ = power, _Rp_ = relative power, _v_ = velocity.

## Data Availability

The data presented in this study are available on request from the corresponding author. The data are not publicly available due to privacy.
